# Performance Development of European Swimmers Across the Olympic Cycle

**DOI:** 10.3389/fspor.2022.894066

**Published:** 2022-06-10

**Authors:** Dennis-Peter Born, Michel Schönfelder, Oliver Logan, Bjørn Harald Olstad, Michael Romann

**Affiliations:** ^1^Swiss Swimming Federation, Section for High-Performance Sports, Bern, Switzerland; ^2^Department for Elite Sport, Swiss Federal Institute of Sport Magglingen, Magglingen, Switzerland; ^3^British Swimming Federation, Sportpark, Loughborough University, Loughborough, United Kingdom; ^4^Department of Physical Performance, Norwegian School of Sport Sciences, Oslo, Norway

**Keywords:** reference values, competition, development, athletes, sports

## Abstract

The aims of the study were to (1) quantify the performance development of race times and key performance indicators of European swimmers across the last Olympic cycle (from 2016 to 2021) and (2) provide reference values for long-course swimming pool events for both sexes from 50 m to 1,500 m including butterfly, backstroke, breaststroke, freestyle, and individual medley. Individual events from the 2016 and 2021 European swimming championships were included. Specifically, 246 men (age: 24.2 ± 3.4 years, FINA points: 890 ± 40) and 256 women races (age: 24.2 ± 4, FINA points: 879 ± 38) of the finalists were recorded and key performance indicators and split times analyzed. Performance differences in finalists of the 2016 and 2021 European championships were determined by an independent *t*-test and Cohen's *d* effect size. Reference values were retrieved from 2021 European championship finalists and are provided for all key performance indicators. Race times improved significantly (*P* < 0.05) or showed moderate (*d* = 0.5–1) to large effect sizes (*d* > 1) in 14 (men) and 6 (women) out of 16 events. Improvements were primarily evident in 100 m and 200 m events for males, as well as BR and sprint events for female swimmers. While start times improved in 15 (men) and 14 (women) events, turn times remained inconclusive in both sexes. Generally, breakout distances increased. Clean swimming velocities were faster in 12 (men) and 5 (women) events. In particular, for alternating swimming strokes, i.e., backstroke and freestyle, effect sizes indicated improved swimming efficiency with an inverse relationship between reduced stroke rate and increased distance per stroke. Coaches and performance analysts may use the present reference values as comparative data for race analyses and to specifically prepare swimmers for the various race sections. Data on the performance development should be used to analyze swimmers' potential and set goals for the various events and the next Olympic cycle.

## Introduction

From a historical perspective, swimming performance has substantially improved since 1960 (Nevill et al., 2007), with women showing an even steeper performance incline than men (Stanula et al., 2012; Sandbakk et al., 2018). After world records plateaued leading up to the 1990s (Nevill et al., 2007), swimming performance has improved slowly but steadily throughout the last decades, in particular in sprint and long-distance events (Konig et al., 2014). The gender-gap in swimming performance has settled at 10% (Nevill et al., 2007; Sandbakk et al., 2018), but decreases the longer the race distance (Wolfrum et al., 2013; Sandbakk et al., 2018). With the continuous development, up-to-date analyses are required to quantify recent performance progression during the Olympic cycle. Detailed race analyses discover potentials and exhibit perspectives for future performance developments specific to sex, race distance, and swimming stroke (Polach et al., 2021; Polach and Born, 2022). In particular, the recent Olympic cycle requires scientific attention. Due to the COVID-19 pandemic, the Olympic Games were postponed, and the Olympic cycle was extended from 4 to 5-years (IOC, 2021). While the longer timeframe increased the window for performance development, COVID-19 lockdowns compromised race times of the top-50 swimmers by 1–2% in the 2019–2020 season (Costa et al., 2021).

However, race section times and key performance indicators may not develop equally to race times. As start performance correlated with the swimmers' strength abilities (West et al., 2011), on-land training routines and strength and conditioning programs are specifically designed to improve the acyclic phases, i.e., start and turn performances (Bishop et al., 2013), which became increasingly important indicators for modern swim races (Morais et al., 2019; Born et al., 2021; Polach et al., 2021). Indeed, the new world record in the men's 1,500 m short-course freestyle (FR) event was broken by improved turns rather than clean swimming performance (Polach et al., 2021; Polach and Born, 2022). Therefore, in addition to total race time, performance development must be assessed for each race section and key performance indicator separately.

At major international competitions, swim analysts closely monitor and analyze races to provide quantitative feedback to their swimmers and coaches (Barbosa et al., 2021). Reference values established by world-class swimmers and championship finalists provide guidelines and benchmarks for individualized case reports (Barbosa et al., 2021). Previous research in the field of race analysis mainly focused on a particular swimming stroke (Gonjo and Olstad, 2020b; Olstad et al., 2020) or race distance (Morais et al., 2019, 2020; Polach et al., 2021) with an underrepresentation of female swimmers (Gonjo and Olstad, 2020a). Therefore, a comprehensive data set with up-to-date reference values for both sexes and long-course pool events from 50 m to 1,500 m including butterfly (BU), backstroke (BA), breaststroke (BR), FR, and individual medley (IM) is warranted.

The aims of the study were to (1) quantify the performance development of race times and key performance indicators in European swimmers across the last Olympic cycle (from 2016 to 2021) and (2) provide reference values for long-course pool events of top elite swimmers for both sexes from 50 m to 1,500 m including BU, BA, BR, FR, and IM.

## Materials and Methods

### Participants

To determine whether key performance indicators and race section times at the European championships provide internationally representative reference values for performance development across the Olympic cycle, race times were extracted from the publicly available database www.swimrankings.net and compared between 2016 and 2021 Olympic games and European championships. To investigate key performance indicators and race section times, individual events from the 2016 and 2021 European long-course swimming championships were included. Specifically, 246 men [mean (minimum–maximum) age: 24.2 ± 3.4 years (17–36), FINA points: 890 ± 40 (771–1,025)] and 256 women in finals [age: 24.2 ± 4 (15–39), FINA points: 879 ± 38 (765–1,005)] were recorded and analyzed for the present study. All participants at the LEN (Ligue Européenne de Natation). European swimming championships agreed to be recorded for television broadcasting and race analysis. The study was approved by the institutional review board of the Swiss Federal Institute of Sport Magglingen (Reg.-Nr. 140_LSP_072021) and is in accordance with the ethical principles for medical research involving human subjects of the World Medical Association (Declaration of Helsinki).

### Data Analysis

Eight stationary cameras recorded the swimmers in each lane individually at a frame rate of 50 Hz and with a panning view. They were placed halfway along the length of the pool, i.e., around the 25 m mark, 20 m away from the pool, and 5 m above the water surface. Video footage of the 2016 European championships was collected using V59 PTZ cameras (Axis Communications AB, Lund, Sweden). High-definition video cameras (2x XAVC S, Sony Group Corporation, Minato, Japan, 5x HC-X1000 and 1x HC-X1500, Panasonic Corporation, Kadoma, Japan) were used for the 2021 European championships. Race times were electronically measured and provided by the official timekeeper, along with the date of birth, which was used to calculate the swimmers' age (Microplus Informatica, Marene CN, Italy).

Race analyses were conducted as described previously (Gonjo and Olstad, 2020a; Barbosa et al., 2021) and video footages were manually analyzed using motion analysis software (Kinovea 0.9.1; Joan Charmant & Contrib., kinovea.org). Video footages were synchronized to the visible light flash of the starting signal and markings at the lane ropes were used to identify the 5, 15, 25, 35, and 45 m marks of each lap. Start times were measured from the starting signal until the top of the head reached the 15 m mark. Turn times were defined as the time from the top of the head reaching the 5 m mark before, to the 15 m mark after the pool wall. As movement velocity progressively reduces during the underwater phase (Gonjo and Olstad, 2020b), breakout distances rather than velocities were measured for a global description of the underwater performance. Breakout distances were measured from the pool wall until the head broke through the water surface. The number of floats on the lane ropes was used to calculate breakout distance to a 10th of a meter. To account for the intra-and inter-lap variability (Simbana-Escobar et al., 2018), stroke rate and distance per stroke were measured twice during each lap. Firstly, at the 15 m mark, but with at least one arm stroke completed after the breakout to limit the interference of transition from the underwater to clean swimming phase (Trinidad et al., 2020), and secondly at the 35 m mark. Stroke rate was determined as 60 divided by stroke time, while distance per stroke was defined as stroke time × section velocity. To compare the results of the present study to previously reported short-course (25 m pool length) data (Olstad et al., 2020), clean swimming velocities rather than times were measured. Clean swimming velocities were determined between the 15 and 45 m mark of each lap. The mean stroke rate, distance per stroke, and clean swimming velocity of each race were used for the statistical analysis. Timestamps tagged in the motion analysis software were imported to a specific spreadsheet to calculate split times for the corresponding key performance indicators (Excel 365, Microsoft Corporation, Redmond, USA).

Missing values due to blocked vision, e.g., by a referee or reflections on the water surface, were replaced by the mean value of that particular final. From a total of 19,254 data points, 12 (0.06%) missing values were treated accordingly. Due to an error in the video recordings of the men's 1,500 m FR final in 2016, only the races of the 3 podium swimmers were available completely, and thus, compared only to the 3 podium swimmers in the 2021 European championships. To ensure inter-rater reliability, 5% of all races were analyzed in duplicate by a second race analyst. Intra-class correlation coefficients with 95% CI showed high reliability for all key performance indicators, i.e., start time 0.995 (0.989–0.998), breakout distance 0.981 (0.957–0.991), turn time 0.998 (0.995–0.999), clean swimming velocity 1 (0.999–1), stroke rate 0.99 (0.978–0.996), and distance per stroke 0.997 (0.992–0.998).

### Statistical Analysis

The statistical analyses were conducted using the statistical software package JASP version.14 (JASP-Team, University of Amsterdam, Amsterdam, The Netherlands). Descriptive data are presented as mean ± *SD*. Development of race times at the Olympic games and European championships across the 5-year Olympic cycle was determined with a 2-way ANOVA: competition (Olympic games vs. European championships) × year (2016 vs. 2021). After confirmation of normality, Tukey's *post-hoc* test was applied, or Bonferroni's test was used if the Levene test showed unequal variances (Field, 2013). Reference values were established based on the mean values of the eight finalists of the particular event at the 2021 European championships. Performance development was assessed and differences between the performance of finalists of the 2016 and 2021 European championships were determined with a Student *t*-test for independent samples with a two-sided alternative hypothesis. If Levene's test showed unequal variances, Welch's *t*-test was used. If the Shapiro-Wilk showed non-normal distributed data, a Mann-Whitney test was conducted. Statistical significance was accepted with an alpha-level ≤ 0.05. To assess results for practical relevance, Cohen's *d* effect sizes (ES) were calculated for the Student and Welch *t*-test (Cohen, 1969; Fröhlich et al., 2009). Effect sizes for the Mann-Whitney test were based on the rank biserial correlation. According to the research population of elite athletes, effect sizes were classified as trivial (<0.25), small (0.25–0.5), moderate (0.5–1), and large (>1), as recommended previously (Fröhlich et al., 2009).

## Results

The number of individuals that qualified for the final of the same event in both the 2016 and 2021 European championships varied from 0 to 4 and was on average 23.44%. Regarding age, the finalists in 2021 were significantly younger in the 100 m BU and FR events compared to 2016. Moderate to large ES indicated older age for 200 m BR, 50 m FR, 400 m FR, and 1,500 m FR. Female finalists were older with moderate ES for 50 m BA, 100 m BA, 100 m BR, and 800 m FR. Descriptive age data for all events are provided in Table 1.

**Table 1:** Age (mean ± standard deviation) of the 2021 European championship finalists, with a significant difference (*) to the 2016 finalists.

	**Age (years)**	
**Event**	**Males**	**Females**
**Butterfly**		
100 m	21.8 ± 2.7 _−1.63_ *	25.0 ± 3.8 _0.44_
200 m	23.1 ± 2.7 _−0.04_	24.4 ± 4.0 _−0.03_
**Backstroke**		
100 m	22.6 ± 2.1 _−0.08_	25.1 ± 3.1 _0.75_
200 m	24.0 ± 3.0 _0.36_	22.3 ± 3.8 _−0.11_
**Breaststroke**		
100 m	24.9 ± 2.4 _0.27_	23.8 ± 4.4 _0.74_
200 m	25.8 ± 2.7 _0.56_	25.3 ± 4.8 _0.42_
**Freestyle**		
50 m	27.5 ± 3.6 _0.50_	27.1 ± 4.4 _0.09_
100 m	20.8 ± 1.9 _−2.70_ *	25.6 ± 5.0 _0.19_
200 m	22.6 ± 3.1 _−0.25_	23.9 ± 4.9 _−0.20_
400 m	23.4 ± 2.8 _0.74_	22.1 ± 4.5 _−0.48_
1,500/800 m	26.0 ± 1.0 _1.00_	24.0 ± 3.4 _0.87_
**Individual medley**		
200 m	25.3 ± 5.3 _0.06_	24.1 ± 5.1 _−0.03_
400 m	24.8 ± 4.7 _0.03_	23.4 ± 4.9 _−0.38_

*Positive and negative effect sizes in _subscript font_ indicate increased or decreased age from 2016 to 2021, respectively*.

Race times significantly improved from 2016 to 2021 European championships in 6 events for males, i.e., 100 m BA, 100 and 200 m BR, 100 and 200 m FR, and 200 m IM (Table 2), but only 2 events for female swimmers, i.e., 100 m BR and 50 m FR (Table 3). In particular, the race times of male European swimmers approximated the Olympic level. While 10 male events in 2016 were significantly faster at the Olympic games than the European championships, this was only the case for 2 events in 2021. In comparison, race times for 9 and 7 female events at the European championships were slower than at the Olympic games in 2016 and 2021, respectively.

**Table 2:** Development of race times in male finalists at the Olympic Games (OG) and European championships (EC) across the 5-year Olympic cycle.

		**Year**					
		**2016**	**2021**		***F***	***P***	** ${\text{ρ}}^{{\text{η}}^{\text{2}}}$ **
**Butterfly**							
100 m	OG EC	00:51.28 ± 00.46 00:51.85 ± 00.64	00:50.65 ± 00.71 00:51.17 ± 00.50	(a)	*F*_(1|28)_ = 10	<0.01	0.26
				(b)	*F*_(1|28)_ = 7	0.01	0.20
				(c)	*F*_(1|28)_ = 0	0.90	0.00
200 m	OG EC	01:54.77 ± 01.40 01:55.85 ± 01.34	01:54.43 ± 01.44 01:54.78 ± 01.73	(a)	*F*_(1|28)_ = 2	0.19	0.06
				(b)	*F*_(1|28)_ = 2	0.19	0.06
				(c)	*F*_(1|28)_ = 0	0.50	0.02
**Backstroke**							
100 m	OG EC	00:52.68 ± 00.54 00:54.20 ± 00.25 #	00:52.44 ± 00.38 00:53.07 ± 00.23 *#	(a)	*F*_(1|28)_ = 28	<0.01	0.50
				(b)	*F*_(1|28)_ = 69	<0.01	0.71
				(c)	*F*_(1|28)_ = 12	<0.01	0.29
200 m	OG EC	01:55.09 ± 01.10 01:58.56 ± 01.87 #	01:55.82 ± 01.84 01:56.59 ± 01.55	(a)	*F*_(1|28)_ = 1	0.29	0.04
				(b)	*F*_(1|28)_ = 14	<0.01	0.33
				(c)	*F*_(1|28)_ = 6	0.03	0.17
**Breaststroke**							
100 m	OG EC	00:58.99 ± 00.84 01:00.33 ± 00.97 #	00:58.60 ± 00.66 00:58.80 ± 00.67 *	(a)	*F*_(1|28)_ = 12	<0.01	0.30
				(b)	*F*_(1|28)_ = 7	0.01	0.21
				(c)	*F*_(1|28)_ = 4	0.05	0.13
200 m	OG EC	02:07.81 ± 00.28 02:10.33 ± 01.29 #	02:07.65 ± 00.84 02:08.54 ± 01.18 *	(a)	*F*_(1|28)_ = 8	0.01	0.22
				(b)	*F*_(1|28)_ = 24	<0.01	0.46
				(c)	*F*_(1|28)_ = 5	0.03	0.16
**Freestyle**							
50 m	OG EC	00:21.67 ± 00.23 00:21.99 ± 00.25 #	00:21.60 ± 00.23 00:21.85 ± 00.19	(a)	*F*_(1|28)_ = 2	0.19	0.06
				(b)	*F*_(1|28)_ = 12	<0.01	0.31
				(c)	*F*_(1|28)_ = 0	0.66	0.01
100 m	OG EC	00:47.96 ± 00.25 00:48.55 ± 00.22 #	00:47.63 ± 00.41 00:47.89 ± 00.38 *	(a)	*F*_(1|28)_ = 18	<0.01	0.39
				(b)	*F*_(1|28)_ = 14	<0.01	0.33
				(c)	*F*_(1|28)_ = 2	0.16	0.07
200 m	OG EC	01:45.47 ± 00.44 01:47.01 ± 00.74 #	01:44.87 ± 00.53 01:45.98 ± 01.02 * #	(a)	*F*_(1|28)_ = 10	<0.01	0.27
				(b)	*F*_(1|28)_ = 27	<0.01	0.49
				(c)	*F*_(1|28)_ = 1	0.41	0.02
400 m	OG EC	03:44.24 ± 02.36 03:47.19 ± 01.72 #	03:44.30 ± 00.76 03:46.05 ± 01.32	(a)	*F*_(1|28)_ = 1	0.36	0.03
				(b)	*F*_(1|28)_ = 16	<0.01	0.37
				(c)	*F*_(1|28)_ = 1	0.31	0.04
1,500 m	OG EC	14:46.81 ± 08.81 14:54.78 ± 10.23	14:50.66 ± 10.33 14:56.52 ± 10.72	(a)	*F*_(1|28)_ = 1	0.44	0.02
				(b)	*F*_(1|28)_ = 4	0.06	0.12
				(c)	*F*_(1|28)_ = 0	0.77	0.00
**Individual medley**							
200 m	OG EC	01:57.13 ± 01.16 02:00.05 ± 00.87 #	01:56.61 ± 01.09 01:57.77 ± 00.68 *	(a)	*F*_(1|28)_ = 17	<0.01	0.37
				(b)	*F*_(1|28)_ = 36	<0.01	0.56
				(c)	*F*_(1|28)_ = 7	0.02	0.19
400 m	OG EC	04:11.22 ± 03.72 04:16.05 ± 02.36 #	04:10.62 ± 00.63 04:13.23 ± 02.59	(a)	*F*_(1|28)_ = 4	0.07	0.11
				(b)	*F*_(1|28)_ = 17	<0.01	0.37
				(c)	*F*_(1|28)_ = 2	0.23	0.05

*(a) Main effect: year (2016 vs. 2021)*.

*(b) Main effect: competition (OG vs. EC)*.

*(c) Interaction effect: year × competition*.

*Significant difference compared to 2021.

*# Significant difference compared to OG*.

*Race times were compared with a 2-way ANOVA: competition (Olympic games vs. European championships) × year (2016 vs. 2021)*.

**Table 3:** Development of race times in female finalists at the Olympic Games (OG) and European championships (EC) across the 5-year Olympic cycle.

		**Year**					
		**2016**	**2021**		***F***	***P***	** ${\text{ρ}}^{{\text{η}}^{\text{2}}}$ **
**Butterfly**							
100 m	OG EC	00:56.63 ± 00.56 00:57.90 ± 01.15 #	00:56.14 ± 00.58 00:57.84 ± 00.40 #	(a)	*F*_(1|27)_ = 1	0.31	0.04
				(b)	*F*_(1|27)_ = 31	<0.01	0.54
				(c)	*F*_(1|27)_ = 1	0.43	0.02
200 m	OG EC	02:06.40 ± 01.32 02:08.49 ± 01.27 #	02:06.78 ± 01.80 02:08.82 ± 01.17 #	(a)	*F*_(1|28)_ = 1	0.48	0.02
				(b)	*F*_(1|28)_ = 17	<0.01	0.38
				(c)	*F*_(1|28)_ = 0	0.96	0.00
**Backstroke**							
100 m	OG EC	00:58.86 ± 00.26 01:00.05 ± 00.92 #	00:58.43 ± 00.69 00:59.59 ± 00.72 #	(a)	*F*_(1|28)_ = 3	0.08	0.10
				(b)	*F*_(1|28)_ = 23	<0.01	0.45
				(c)	*F*_(1|28)_ = 0	0.96	0.00
200 m	OG EC	02:07.84 ± 01.29 02:10.11 ± 02.17	02:06.75 ± 01.42 02:09.10 ± 02.11	(a)	*F*_(1|28)_ = 3	0.11	0.09
				(b)	*F*_(1|28)_ = 13	<0.01	0.32
				(c)	*F*_(1|28)_ = 0	0.95	0.00
**Breaststroke**							
100 m	OG EC	01:06.47 ± 01.06 01:07.43 ± 00.71	01:05.85 ± 00.62 01:06.32 ± 00.33 *	(a)	*F*_(1|28)_ = 11	<0.01	0.29
				(b)	*F*_(1|28)_ = 8	0.01	0.21
				(c)	*F*_(1|28)_ = 1	0.34	0.03
200 m	OG EC	02:22.37 ± 01.01 02:24.16 ± 02.47	02:21.70 ± 01.91 02:23.29 ± 01.76	(a)	*F*_(1|28)_ = 1	0.25	0.05
				(b)	*F*_(1|28)_ = 7	0.02	0.19
				(c)	*F*_(1|28)_ = 0	0.89	0.00
**Freestyle**							
50 m	OG EC	00:24.23 ± 00.22 00:24.79 ± 00.39 #	00:24.23 ± 00.20 00:24.34 ± 00.25 *	(a)	*F*_(1|28)_ = 5	0.03	0.16
				(b)	*F*_(1|28)_ = 12	<0.01	0.31
				(c)	*F*_(1|28)_ = 5	0.03	0.16
100 m	OG EC	00:53.05 ± 00.25 00:54.41 ± 01.20 #	00:52.61 ± 00.38 00:53.55 ± 00.33 #	(a)	*F*_(1|28)_ = 8	0.01	0.22
				(b)	*F*_(1|28)_ = 24	<0.01	0.46
				(c)	*F*_(1|28)_ = 1	0.38	0.03
200 m	OG EC	01:55.12 ± 00.91 01:57.48 ± 01.14 #	01:55.01 ± 00.96 01:57.61 ± 01.34 #	(a)	*F*_(1|28)_ = 0	0.98	0.00
				(b)	*F*_(1|28)_ = 41	<0.01	0.59
				(c)	*F*_(1|28)_ = 0	0.76	0.00
400 m	OG EC	04:03.08 ± 03.35 04:07.63 ± 02.35 #	04:02.68 ± 04.13 04:07.99 ± 02.33 #	(a)	*F*_(1|28)_ = 0	0.99	0.00
				(b)	*F*_(1|28)_ = 20	<0.01	0.41
				(c)	*F*_(1|28)_ = 0	0.73	0.00
800 m	OG EC	08:18.68 ± 06.85 08:28.48 ± 04.91 #	08:19.90 ± 04.86 08:28.12 ± 05.10 #	(a)	*F*_(1|28)_ = 0	0.82	0.00
				(b)	*F*_(1|28)_ = 22	<0.01	0.43
				(c)	*F*_(1|28)_ = 0	0.69	0.01
**Individual medley**							
200 m	OG EC	02:09.93 ± 02.50 02:11.69 ± 02.28	02:10.03 ± 01.68 02:11.11 ± 01.21	(a)	*F*_(1|28)_ = 0	0.74	0.00
				(b)	*F*_(1|28)_ = 4	0.05	0.13
				(c)	*F*_(1|28)_ = 0	0.63	0.01
400 m	OG EC	04:33.15 ± 03.47 04:39.12 ± 04.22 #	04:35.94 ± 02.87 04:39.96 ± 04.15	(a)	*F*_(1|28)_ = 2	0.18	0.06
				(b)	*F*_(1|28)_ = 14	<0.01	0.34
				(c)	*F*_(1|28)_ = 1	0.46	0.02

*(a) Main effect: year (2016 vs. 2021)*.

*(b) Main effect: competition (OG vs. EC)*.

*(c) Interaction effect: year × competition*.

*Significant difference compared to 2021.

*# Significant difference compared to OG*.

*Race times were compared with a 2-way ANOVA: competition (Olympic games vs. European championships) × year (2016 vs. 2021)*.

Reference values for race times and key performance indicators were retrieved from the 2021 European championship finalists and are presented with the performance development across the Olympic cycle in Table 4 (men) and Table 5 (women). Descriptive data (mean ± standard deviation) for both European championships, including effect sizes with 95% CIs, *P*-values, and %-differences for performance development from 2016 to 2021 are provided in Supplementary Tables 1–8.

**Table 4:** Reference values (mean ± standard deviation) for key performance indicators retrieved from male 2021 European championship finalists with significant difference (*) to the 2016 finalists.

**Event**	**Start time (s)**	**Breakout distance** **after start (m)**	**Turn time (s)**	**Breakout distance** **after turn (m)**	**Clean swimming** **velocity (m/s)**	**Stroke rate** **(bpm)**	**Distance per** **stroke (m)**
**Butterfly**							
100 m	5.53 ± 0.19_1.54_*	13.4 ± 0.4_0.28_	10.39 ± 0.13_0.06_	11.7 ± 1.5_0.38_	1.84 ± 0.02_0.95_	56.1 ± 2.6_0.86_	1.98 ± 0.11_−0.38_
200 m	5.91 ± 0.08_2.07_*	13.5 ± 0.3_0.78_	11.55 ± 0.18_−0.39_	9.8 ± 1.3_0.62_	1.68 ± 0.03_0.53_	49.8 ± 1.8_0.26_	2.03 ± 0.10_−0.05_
**Backstroke**							
100 m	6.20 ± 0.17_1.33_*	13.3 ± 0.7_−0.79_	10.15 ± 0.16_1.07_*	11.5 ± 1.1_0.30_	1.77 ± 0.02_1.92_*	49.7 ± 3.4_−0.22_	2.15 ± 0.14_0.60_
200 m	6.46 ± 0.23_0.73_	13.6 ± 0.6_0.14_	10.99 ± 0.33_0.68_	11.4 ± 2.2_0.92_	1.62 ± 0.03_1.00_	41.4 ± 2.5_−0.50_	2.35 ± 0.12_0.82_
**Breaststroke**							
100 m	6.43 ± 0.25_1.50_*	14.0 ± 0.7_0.50_	11.77 ± 0.11_1.22_*	10.5 ± 0.9_0.97_	1.60 ± 0.02_1.38_*	55.2 ± 3.7_0.68_	1.75 ± 0.11_−0.45_
200 m	6.47 ± 0.36_0.81_	15.5 ± 1.7_0.42_	12.55 ± 0.20_−0.25_	10.8 ± 1.0_0.42_	1.48 ± 0.02_1.55_*	35.7 ± 3.1_−0.39_	2.51 ± 0.21_0.76_
**Freestyle**							
50 m	5.31 ± 0.11_1.33_*	10.4 ± 1.7_0.35_			2.12 ± 0.02_−0.39_	61.9 ± 1.8_−0.45_	2.05 ± 0.06_0.41_
100 m	5.55 ± 0.14_2.02_*	11.9 ± 0.7_0.55_	9.51 ± 0.19_−0.60_	8.1 ± 1.7_0.53_	1.98 ± 0.02_1.55_*	50.9 ± 2.4_−0.99_	2.34 ± 0.10_1.43_*
200 m	5.84 ± 0.16_0.92_	12.3 ± 0.6_0.76_	10.39 ± 0.18_−0.33_	7.4 ± 1.3_1.47_*	1.81 ± 0.02_0.66_*	43.4 ± 2.0_−0.31_	2.51 ± 0.12_0.64_
400 m	6.07 ± 0.16_1.31_*	11.3 ± 2.0_0.22_	10.87 ± 0.13_−0.59_	6.8 ± 0.5_1.53_*	1.70 ± 0.01_1.05_*	39.5 ± 2.8_−1.26_*	2.60 ± 0.17_1.47_*
1,500 m	6.67 ± 0.06_0.17_	10.6 ± 1.1_0.05_	11.37 ± 0.11_−0.81_	5.1 ± 1.1_−0.33_	1.65 ± 0.01_0.00_	37.8 ± 5.1_−0.34_	2.64 ± 0.36_0.37_
**Individual medley**							
200 m	5.84 ± 0.15_1.23_*	12.7 ± 0.5_−1.02_	11.80 ± 0.22_−0.24_	9.6 ± 0.9_0.05_	1.63 ± 0.02_2.85_*	44.3 ± 1.7_0.22_	2.22 ± 0.10_0.48_
400 m	6.26 ± 0.19_1.01_	12.7 ± 0.9_0.28_	12.43 ± 0.18_−0.07_	8.2 ± 0.9_0.03_	1.53 ± 0.02_1.28_*	40.5 ± 1.5_−0.28_	2.27 ± 0.09_0.60_

*Positive and negative effect sizes in _subscript font_ indicate improved or decreased performance, respectively*.

**Table 5:** Reference values (mean ± standard deviation) for key performance indicators retrieved from female 2021 European championship finalists with significant difference (*) to the 2016 finalists.

**Event**	**Start time (s)**	**Breakout distance** **(m) after start**	**Turn time (s)**	**Breakout distance** **(m) after turn**	**Clean swimming** **velocity (m/s)**	**Stroke rate** **(bpm)**	**Distance per** **stroke (m)**
**Butterfly**							
100 m	6.33 ± 0.18_2.02_*	13.2 ± 1.0_0.94_	11.63 ± 0.25_−0.08_	9.7 ± 2.2_0.83_	1.63 ± 0.02_−0.48_	56.4 ± 3.0_0.23_	1.74 ± 0.10_−0.37_
200 m	6.96 ± 0.19_0.54_	12.0 ± 1.1_0.06_	12.88 ± 0.16_−0.95_	8.0 ± 1.5_0.25_	1.50 ± 0.02_0.07_	51.6 ± 2.5_0.07_	1.75 ± 0.09_−0.07_
**Backstroke**							
100 m	7.09 ± 0.20_0.69_*	13.4 ± 0.5_0.41_	11.48 ± 0.29_−0.36_	10.0 ± 2.1_−0.34_	1.58 ± 0.04_0.07_	48.1 ± 3.4_−0.65_	1.97 ± 0.15_0.65_
200 m	7.61 ± 0.24_0.00_	13.0 ± 0.7_0.16_	12.43 ± 0.22_−0.30_	8.5 ± 1.6_0.52_	1.48 ± 0.03_0.90_	41.1 ± 4.0_−0.87_	2.18 ± 0.20_1.18_*
**Breaststroke**							
100 m	7.55 ± 0.25_0.69_*	12.5 ± 0.5_1.10_*	13.37 ± 0.16_−0.12_	8.7 ± 0.5_0.90_	1.43 ± 0.01_0.88_*	48.6 ± 6.1_0.31_	1.79 ± 0.22_−0.07_
200 m	7.83 ± 0.15_1.27_*	13.1 ± 0.8_0.84_*	14.05 ± 0.24_−0.28_	9.2 ± 0.6_2.04_*	1.34 ± 0.02_0.28_	35.8 ± 3.8_−0.21_	2.27 ± 0.24_0.32_
**Freestyle**							
50 m	6.01 ± 0.17_1.58_*	11.8 ± 0.9_0.81_			1.91 ± 0.02_0.66_	60.2 ± 2.9_−0.18_	1.91 ± 0.08_0.40_
100 m	6.25 ± 0.20_1.61_*	11.8 ± 1.1_0.89_	10.62 ± 0.15_0.19_	7.2 ± 1.3_0.87_	1.77 ± 0.02_0.71_	49.1 ± 2.8_−0.42_	2.17 ± 0.13_0.57_
200 m	6.65 ± 0.17_1.82_*	10.5 ± 0.7_0.99_	11.51 ± 0.16_−0.40_	5.4 ± 0.8_1.03_	1.64 ± 0.02_−0.20_	43.7 ± 2.8_−0.41_	2.25 ± 0.14_0.36_
400 m	7.15 ± 0.23_0.67_	9.94 ± 1.1_0.04_	12.11 ± 0.14_−1.21_*	3.9 ± 0.7_−0.63_	1.57 ± 0.02_0.42_	45.2 ± 4.8_0.32_	2.10 ± 0.22_−0.19_
800 m	7.23 ± 0.22_1.76_*	9.73 ± 0.9_0.46_	12.21 ± 0.16_−0.12_	4.0 ± 0.5_−0.76_	1.53 ± 0.02_0.08_	44.8 ± 3.8_0.36_	2.06 ± 0.16_−0.33_
**Individual medley**							
200 m	6.74 ± 0.24_0.66_	12.3 ± 1.1_0.75_	13.17 ± 0.23_0.03_	7.2 ± 1.1_0.41_	1.47 ± 0.01_0.38_	45.2 ± 2.4_−0.02_	1.96 ± 0.11_0.10_
400 m	7.08 ± 0.28_0.45_	12.0 ± 1.1_0.54_	13.76 ± 0.26_−0.47_	6.2 ± 0.4_−0.33_	1.39 ± 0.02_0.23_	42.0 ± 2.3_−0.10_	1.99 ± 0.09_0.15_

*Positive and negative effect sizes in _subscript font_ indicate improved or decreased performance, respectively*.

Regarding performance development across the 5-year timespan, start times improved (in 15 events for men and 14 events for women), while turn-times remained inconclusive for both sexes. Generally, breakout distances increased. Clean swimming velocities became faster (in 12 events for men and 5 events for women). ES indicated an inverse relationship between reduced stroke rate and increased distance per stroke, in particular for alternating swimming strokes, i.e., BA and FR.

## Discussion

The present study provides reference values for long-course swimming pool events for both sexes from 50 to 1,500 m including BU, BA, BR, FR, and IM. Descriptive data (mean ± *SD*) and %-differences between both the 2016 and 2021 European championships are provided in Supplementary Material and may be used for practical assessment of performance development. In particular, male European swimmers approximated the performance level of the Olympic games and significantly improved race times across the 5-year timespan in 100 m BA, 100 m and 200 m BR, 100 m and 200 m FR, and 200 m IM. Performance development was less pronounced in female swimmers, where race times only improved significantly for 100 m BR and 50 m FR. A comparison of key performance indicators showed significantly improved start times with medium to large ES for longer breakout distances. Furthermore, clean swimming velocities improved, while changes in turn performances remained inconclusive. Effect sizes indicated an inverse relationship between reduced stroke rate and increased distance per stroke in particular for alternating swimming strokes, i.e., BA and FR.

Due to limited accreditations for race analysts and scientists at the Olympic games, the present study determined benchmarks for key performance indicators from races at the European swimming championships. To relate performance data of European swimmers to the Olympic level, the development of race times across the 5-year timeframe was compared between the European championships and Olympic games. The results show that male European swimmers caught up with the world-class swimmers across the recent Olympic cycle, as only 2 events in 2021, compared to 10 in 2016, were significantly slower than in the Olympic finals. Therefore, key performance indicators and race section times can be used as benchmarks for a broad range of international male swimmers. In contrast, in 2021 female European swimmers still showed significantly slower race times in 7 events, thus, the actual race time needs to be considered when using the reference values for international female swimmers.

In the present study, start times improved across the 5-year Olympic cycle. The start involves the on-block, flight, underwater, and transition to clean swimming phases (Tor et al., 2015b). In particular, during phases above water, i.e., block and flight phases, previous studies found that start performance was related to the horizontal take-off velocity (Tor et al., 2015b) and swimmers' maximal strength abilities (West et al., 2011). Multiple studies have shown that particularly the acyclic phases, i.e., start performance, profit from various strength training methods (Bishop et al., 2013; Thng et al., 2019). However, on-block force production could not be investigated with the video-based race analyses, and altered strength abilities and conditioning regimes that may have improved start performance across the recent years are a matter of future studies. Yet, the present study showed medium to large ES for improved breakout distances in addition to the improved start performances across the Olympic cycle. While the present study is the first to report competition-based breakout distances for IM to the best of our knowledge, the underwater phase is a critical race section, as the momentum gained from the push-off transitions to the clean swimming phase (Veiga and Roig, 2017; Olstad et al., 2020). As drag forces are lower underwater than at the surface (Tor et al., 2015a), swimmers aim to extend the underwater phase to the regulatory limits of 15 m (FINA, 2022). Previously reported underwater distances were shorter (13.1 ± 1 m) (Olstad et al., 2020) compared to the present study (14 ± 0.7 m). However, the performance level of the swimmers in previous studies was lower (688 ± 87 FINA points) compared to the present European championship finalists (890 ± 40 FINA points). Therefore, higher-ranked swimmers achieved faster underwater velocities and underwater distances were related to swimming performance (Pla et al., 2021). However, lack of oxygen due to restricted breathing results in apnea-induced discomfort (Veiga et al., 2022). In order to not interfere with transition and clean swimming phases, the length of the underwater phase should match an individual's optimum rather than maximum (Veiga et al., 2022). Specific breathing maneuvers and apnea training regimes may help to achieve longer underwater phases (Woorons et al., 2016; Robertson et al., 2020).

Improved race times were accompanied by faster clean swimming velocities, rather than turn performances, this was particularly noteworthy for middle-distance events. In practice, turns are performed repeatedly between every lap. However, with most training sessions performed at low intensities (Pollock et al., 2019), most turns are performed with slower velocities than during competition. In contrast, speed training referred to as alactic sprints by practitioners, is typically performed from 15 to 25 m after pushing off the pool wall or from the starting block (Pollock et al., 2019). However, turns require a complex set of specific technical skills, i.e., the timing of wall approach, rotation, push-off, gliding, underwater kicking, and transition to full-stroke swimming (Olstad et al., 2020; Nicol et al., 2021). The complexity of these performance indicators contributing to total turn time underlines the importance of race pace-specific turn drills implemented in speed training sessions. More research focusing on turn performance may enhance awareness and focus in practices on this particular part of the swim race. Thus, turn performance may allow for future performance developments, such as in the men's 1,500 m FR short-course event in which turn performance was the distinguishing factor for World championship finalists and a new World record (Polach et al., 2021; Polach and Born, 2022). Additionally, recent developments of new competition formats, i.e., the International Swimming League (ISL), which emphasizes short-course and sprint races, may further contribute to the development of the acyclic phases (ISL, 2022).

Generally, clean swimming velocity results from the product of stroke rate and stroke length. Across the Olympic cycle, effect sizes showed an improved swimming efficiency with decreased stroke rate and increased distance per stroke, in particular for the alternating swimming strokes, i.e., BA and FR. Previous studies showed the importance of on-land conditioning regimes to develop the required maximal strength for improvements in stroke length (Crowley et al., 2017). Traditional training methods, such as swimming with hand paddles and resisted in-water drills, have a larger effect on stroke rate and help to transfer strength gains to the sport-specific movement (Girold et al., 2006; Crowley et al., 2017). Therefore, current development toward higher awareness and implementation of on-land strength and conditioning and weight-lifting routines in elite swimmers (Crowley et al., 2018; Nugent et al., 2018; Pollock et al., 2019) may explain the increased distance per stroke across the Olympic cycle found in the present study.

In the present study, the age of European championship finalists was 20.8–27.5 years for males and 22.1–27.1 years for female swimmers. This is in line with previous findings showing that the age of peak performance varies between 21.4 and 26.1 years depending on the event (Allen et al., 2014). Based on race results, previous studies used longitudinal tracking to establish individual trajectories for performance development (Pyne et al., 2004; Post et al., 2020). By accounting for drop-out, the value of longitudinal tracking is clear (Born et al., 2022). However, the present study aimed to investigate the development of key performance indicators and race section times with video-based race analyses across an Olympic cycle. The number of individuals qualifying for the final of the same event in both the 2016 and 2021 European championships varied from 0 to 4 and was on average as low as 23.44%. This limited the longitudinal comparison of individual performance trajectories across the 5-year timeframe. To avoid the number of subjects randomly confounding findings due to lower statistical power in events with low sample size, all finalists of each event were therefore included in the cross-sectional analysis. Future studies should investigate individual pathways of the development of key performance indicators and race section times across a longer timeframe, i.e., from junior to elite age or even across the entire swimming career. With continuous data collection of video footage at future swimming competitions, i.e., European championships, such longitudinal studies will be possible by the end of the current Olympic cycle (2024). Additionally, multiple milestones, i.e., European championships in Glasgow 2018 and Rome 2022, could be added for a more detailed investigation of performance progression.

## Conclusion

For the present study, up-to-date reference values of key performance indicators for long-course pool swimming events were retrieved from finalists at the 2021 European championship. In particular, male European swimmers approximated performance levels at the Olympic games and showed significantly improved races times in 100 m BA, 100 m and 200 m BR, 100 m and 200 m FR, and 200 m IM from 2016 to 2021. In 2021, only 2 European finals, compared to 10 in 2016, were significantly slower than the Olympic finals. Female European swimmers significantly improved in only 2 events across the 5-year timespan and remained slower in 7 events compared to Olympic finalists. Analysis of key performance indicators showed improved start performances, longer breakout distances, and improved clean swimming velocities. Effect sizes indicated reduced stroke rate in alternating swimming strokes, i.e., BA and FR, with an increase in distance per stroke. Coaches and performance analysts may use the present reference values as comparative data for race analyses and to specifically prepare for the various race sections. Data on the performance development should be used to analyze swimmers' potential and set goals for the various events and the next Olympic cycle.

## Data Availability Statement

The original contributions presented in the study are included in the article/Supplementary Material, further inquiries can be directed to the corresponding author.

## Ethics Statement

The studies involving human participants were reviewed and approved by Swiss Federal Institute of Sport Magglingen. Written informed consent for participation was not required for this study in accordance with the national legislation and the institutional requirements.

## Author Contributions

The conception of the experimental design: D-PB and MR. Data collection and data analysis: D-PB, MS, OL, and MR. Data interpretation: D-PB, OL, BO, and MR. Preparing the manuscript: D-PB. Critically revising the manuscript: MS, OL, BO, and MR. Reading and approving final version of the manuscript: D-PB, MS, OL, BO, and MR. All authors contributed to the article and approved the submitted version.

## Funding

The study was financed by the research fund of the National Sports Association (Swiss Olympics). The funder had no role in the conception of study design, data collection, data analysis, interpretation of the data, writing of the report, or decision to submit the article for publication.

## Conflict of Interest

The authors declare that the research was conducted in the absence of any commercial or financial relationships that could be construed as a potential conflict of interest.

## Publisher's Note

All claims expressed in this article are solely those of the authors and do not necessarily represent those of their affiliated organizations, or those of the publisher, the editors and the reviewers. Any product that may be evaluated in this article, or claim that may be made by its manufacturer, is not guaranteed or endorsed by the publisher.
