# Supplementary material for: Performance Development of European Swimmers Across the Olympic Cycle
Source: Front Sports Act Living. 2022 Jun 10;4:894066. doi: 10.3389/fspor.2022.894066 (PMC9231649; doi:10.3389/fspor.2022.894066)
Supplement: Supplementary file 1 [file Data_Sheet_1.pdf]

Supplementary Table 1. Comparing age [years] between the 2016 and 2021 European championship finalists using independent t-test, effect size (ES) with 95% confidence interval (CI), and percent difference (%-diff).

Event	Competition year		*P*	ES [95% CI]	%-diff
	2016	2021			
**Males**					
**Butterfly**					
100 m	26.5 ± 3.1	21.8 ± 2.7	0.01	-1.63 [-2.75- -0.46]	-17.74
200 m	23.3 ± 3.9	23.1 ± 2.7	0.94	-0.04 [-1.02-0.94]	-0.86
**Backstroke**					
100 m	22.9 ± 4.0	22.6 ± 2.1	0.88	-0.08 [-1.06-0.90]	-1.31
200 m	23.0 ± 2.5	24.0 ± 3.0	0.48	0.36 [-0.64-1.34]	4.35
**Breaststroke**					
100 m	24.3 ± 2.3	24.9 ± 2.4	0.60	0.27 [-0.72-1.25]	2.47
200 m	23.4 ± 2.1	25.8 ± 2.7	0.06	0.56 [0.05-0.84]	10.26
**Freestyle**					
50 m	25.9 ± 2.9	27.5 ± 3.6	0.34	0.50 [-0.51-1.49]	6.18
100 m	26.5 ± 2.3	20.8 ± 1.9	<0.01	-2.70 [-4.07- -1.28]	-21.51
200 m	23.4 ± 2.8	22.6 ± 3.1	0.62	-0.25 [-1.23-0.73]	-3.42
400 m	21.8 ± 1.4	23.4 ± 2.8	0.17	0.74 [-0.30-1.75]	7.34
1500 m	21.3 ± 1.2	26.0 ± 1.0	0.08	1.00 [1.00-1.00]	22.07
**Individual medley**					
200 m	25.0 ± 3.2	25.3 ± 5.3	0.91	0.06 [-0.92-1.04]	1.20
400 m	24.6 ± 3.6	24.8 ± 4.7	0.96	0.03 [-0.50-0.55]	0.81
**Females**					
**Butterfly**					
100 m	23.4 ± 3.6	25.0 ± 3.8	0.39	0.44 [-0.56-1.43]	6.84
200 m	24.5 ± 3.1	24.4 ± 4.0	0.95	-0.03 [-1.01-0.95]	-0.41
**Backstroke**					
100 m	22.9 ± 2.9	25.1 ± 3.1	0.16	0.75 [-0.28-1.76]	9.61
200 m	22.8 ± 2.3	22.3 ± 3.8	0.75	-0.11 [-0.60-0.44]	-2.19
**Breaststroke**					
100 m	21.0 ± 2.9	23.8 ± 4.4	0.16	0.74 [-0.29-1.74]	13.33
200 m	23.4 ± 4.1	25.3 ± 4.8	0.41	0.42 [-0.58-1.41]	8.12
**Freestyle**					
50 m	27.1 ± 5.2	27.1 ± 4.4	0.79	0.09 [-0.45-0.59]	0.00
100 m	24.9 ± 2.6	25.6 ± 5.0	0.71	0.19 [-0.80-1.17]	2.81
200 m	24.6 ± 4.6	23.9 ± 4.9	0.52	-0.20 [-0.66-0.36]	-2.85
400 m	23.9 ± 2.5	22.1 ± 4.5	0.35	-0.48 [-1.47-0.52]	-7.53
800 m	21.4 ± 2.6	24.0 ± 3.4	0.10	0.87 [-0.18-1.89]	12.15
**Individual medley**					
200 m	24.4 ± 4.1	24.1 ± 5.1	0.96	-0.03 [-0.55-0.50]	-1.23
400 m	24.9 ± 2.6	23.4 ± 4.9	0.46	-0.38 [-1.36-0.62]	-6.02

Supplementary Table 2. Comparing start time [s] between the 2016 and 2021 European championships using independent t-test, effect size (ES) with 95% confidence interval (CI), and percent difference (%-diff).

Event	Competition year		*P*	ES [95% CI]	%-diff
	2016	2021			
**Males**					
**Butterfly**					
100 m	5.79 ± 0.14	5.53 ± 0.19	<0.01	1.54 [0.39-2.65]	4.49
200 m	6.18 ± 0.17	5.91 ± 0.08	<0.01	2.07 [0.81-3.29]	4.37
**Backstroke**					
100 m	6.38 ± 0.09	6.20 ± 0.17	0.02	1.33 [0.22-2.41]	2.82
200 m	6.64 ± 0.26	6.46 ± 0.23	0.16	0.73 [-0.29-1.74]	2.71
**Breaststroke**					
100 m	6.79 ± 0.23	6.43 ± 0.25	<0.01	1.50 [0.36-2.60]	5.30
200 m	6.72 ± 0.24	6.47 ± 0.36	0.13	0.81 [-0.23-1.82]	3.72
**Freestyle**					
50 m	5.49 ± 0.16	5.31 ± 0.11	0.02	1.33 [0.21-2.40]	3.28
100 m	5.83 ± 0.15	5.55 ± 0.14	<0.01	2.02 [0.77-3.23]	4.80
200 m	6.01 ± 0.21	5.84 ± 0.16	0.09	0.92 [-0.13-1.94]	2.83
400 m	6.31 ± 0.21	6.07 ± 0.16	0.02	1.31 [0.20-2.38]	3.80
1500 m	6.71 ± 0.27	6.67 ± 0.06	0.85	0.17 [-1.46-1.76]	0.60
**Individual medley**					
200 m	6.07 ± 0.21	5.84 ± 0.15	0.03	1.23 [0.14-2.30]	3.79
400 m	6.45 ± 0.18	6.26 ± 0.19	0.06	1.01 [-0.05-2.04]	2.95
**Females**					
**Butterfly**					
100 m	6.75 ± 0.24	6.33 ± 0.18	<0.01	2.02 [0.77-3.22]	6.22
200 m	7.09 ± 0.26	6.96 ± 0.19	0.30	0.54 [-0.47-1.53]	1.83
**Backstroke**					
100 m	7.39 ± 0.26	7.09 ± 0.20	0.02	0.69 [0.25-0.89]	4.06
200 m	7.61 ± 0.43	7.61 ± 0.24	1.00	0.00 [-0.98-0.98]	0.00
**Breaststroke**					
100 m	7.82 ± 0.26	7.55 ± 0.25	0.02	0.69 [0.25-0.89]	3.45
200 m	8.07 ± 0.21	7.83 ± 0.15	0.02	1.27 [0.17-2.33]	2.97
**Freestyle**					
50 m	6.30 ± 0.20	6.01 ± 0.17	<0.01	1.58 [0.42-2.70]	4.60
100 m	6.66 ± 0.30	6.25 ± 0.20	<0.01	1.61 [0.44-2.73]	6.16
200 m	6.97 ± 0.18	6.65 ± 0.17	<0.01	1.82 [0.61-2.99]	4.59
400 m	7.29 ± 0.19	7.15 ± 0.23	0.20	0.67 [-0.35-1.67]	1.92
800 m	7.58 ± 0.17	7.23 ± 0.22	<0.01	1.76 [0.56-2.91]	4.62
**Individual medley**					
200 m	6.94 ± 0.34	6.74 ± 0.24	0.21	0.66 [-0.36-1.65]	2.88
400 m	7.20 ± 0.22	7.08 ± 0.28	0.38	0.45 [-0.55-1.44]	1.67

Supplementary Table 3. Comparing breakout distance after the start [m] between the 2016 and 2021 European championships using independent t-test, effect size (ES) with 95% confidence interval (CI), and percent difference (%-diff).

Event	Competition year		*P*	ES [95% CI]	%-diff
	2016	2021			
**Males**					
**Butterfly**					
100 m	13.28 ± 0.86	13.46 ± 0.36	0.58	0.28 [-0.71-1.26]	1.36
200 m	12.86 ± 1.13	13.51 ± 0.34	0.15	0.78 [-0.29-1.81]	5.05
**Backstroke**					
100 m	13.86 ± 0.66	13.33 ± 0.69	0.14	-0.79 [-1.80-0.25]	-3.82
200 m	13.28 ± 0.92	13.69 ± 0.63	0.67	0.14 [-0.42-0.62]	3.09
**Breaststroke**					
100 m	13.52 ± 0.73	14.09 ± 0.72	0.10	0.50 [-0.03-0.81]	4.22
200 m	14.87 ± 1.25	15.50 ± 1.73	0.42	0.42 [-0.58-1.40]	4.24
**Freestyle**					
50 m	9.89 ± 1.52	10.45 ± 1.71	0.50	0.35 [-0.65-1.33]	5.66
100 m	11.15 ± 1.82	11.91 ± 0.74	0.30	0.55 [-0.48-1.54]	6.82
200 m	11.63 ± 1.16	12.33 ± 0.58	0.16	0.76 [-0.29-1.77]	6.02
400 m	11.35 ± 0.82	11.34 ± 1.96	0.49	0.22 [-0.35-0.67]	-0.09
1500 m	10.53 ± 1.65	10.60 ± 1.06	0.96	0.05 [-1.56-1.65]	0.66
**Individual medley**					
200 m	13.49 ± 0.81	12.78 ± 0.54	0.06	-1.02 [-2.05-0.04]	-5.26
400 m	12.41 ± 1.03	12.78 ± 0.86	0.37	0.28 [-0.29-0.70]	2.98
**Females**					
**Butterfly**					
100 m	12.17 ± 1.27	13.25 ± 1.00	0.08	0.94 [-0.11-1.97]	8.87
200 m	11.98 ± 1.35	12.05 ± 1.09	0.91	0.06 [-0.92-1.04]	0.58
**Backstroke**					
100 m	13.00 ± 1.51	13.46 ± 0.52	0.43	0.41 [-0.59-1.39]	3.54
200 m	12.88 ± 1.73	13.09 ± 0.68	0.65	0.16 [-0.63-0.40]	1.63
**Breaststroke**					
100 m	11.91 ± 0.67	12.55 ± 0.48	0.05	1.10 [0.02-2.14]	5.37
200 m	12.22 ± 0.68	13.15 ± 0.81	<0.01	0.84 [0.57-0.95]	7.61
**Freestyle**					
50 m	10.89 ± 1.41	11.84 ± 0.86	0.13	0.81 [-0.22-1.82]	8.72
100 m	10.93 ± 0.97	11.85 ± 1.10	0.10	0.89 [-0.16-1.91]	8.42
200 m	9.95 ± 0.52	10.56 ± 0.70	0.07	0.99 [-0.07-2.02]	6.13
400 m	9.88 ± 1.70	9.94 ± 1.13	0.93	0.04 [-0.94-1.02]	0.61
800 m	9.26 ± 1.10	9.73 ± 0.94	0.37	0.46 [-0.54-1.45]	5.08
**Individual medley**					
200 m	11.47 ± 1.29	12.38 ± 1.12	0.16	0.75 [-0.28-1.75]	7.93
400 m	11.47 ± 1.18	12.08 ± 1.07	0.30	0.54 [-0.47-1.53]	5.32

Supplementary Table 4. Comparing turn time [s] between the 2016 and 2021 European championships using independent t-test, effect size (ES) with 95% confidence interval (CI), and percent difference (%-diff).

Event	Competition year		*P*	ES [95% CI]	%-diff
	2016	2021			
**Males**					
**Butterfly**					
100 m	10.40 ± 0.29	10.39 ± 0.13	0.91	0.06 [-0.93-1.03]	0.10
200 m	11.48 ± 0.20	11.55 ± 0.18	0.45	-0.39 [-1.37-0.61]	-0.61
**Backstroke**					
100 m	10.33 ± 0.17	10.15 ± 0.16	0.05	1.07 [0.00-2.11]	1.74
200 m	11.23 ± 0.35	10.99 ± 0.33	0.19	0.68 [-0.34-1.68]	2.14
**Breaststroke**					
100 m	11.94 ± 0.17	11.77 ± 0.11	0.03	1.22 [0.12-2.28]	1.42
200 m	12.51 ± 0.12	12.55 ± 0.20	0.62	-0.25 [-1.23-0.74]	-0.32
**Freestyle**					
100 m	9.41 ± 0.13	9.51 ± 0.19	0.25	-0.60 [-1.59-0.41]	-1.06
200 m	10.34 ± 0.11	10.39 ± 0.18	0.29	-0.33 [-0.73-0.24]	-0.48
400 m	10.81 ± 0.06	10.87 ± 0.13	0.26	-0.59 [-1.59-0.43]	-0.56
1500 m	11.30 ± 0.07	11.37 ± 0.11	0.38	-0.81 [-2.46-0.92]	-0.62
**Individual medley**					
200 m	11.75 ± 0.16	11.80 ± 0.22	0.63	-0.24 [-1.22-0.74]	-0.43
400 m	12.42 ± 0.12	12.43 ± 0.18	0.90	-0.07 [-1.04-0.92]	-0.08
**Females**					
**Butterfly**					
100 m	11.61 ± 0.25	11.63 ± 0.25	0.88	-0.08 [-1.06-0.90]	-0.17
200 m	12.70 ± 0.21	12.88 ± 0.16	0.08	-0.95 [-1.97-0.11]	-1.42
**Backstroke**					
100 m	11.37 ± 0.29	11.48 ± 0.29	0.48	-0.36 [-1.35-0.63]	-0.97
200 m	12.34 ± 0.34	12.43 ± 0.22	0.55	-0.30 [-1.28-0.69]	-0.73
**Breaststroke**					
100 m	13.34 ± 0.25	13.37 ± 0.16	0.81	-0.12 [-1.10-0.86]	-0.22
200 m	13.97 ± 0.28	14.05 ± 0.24	0.59	-0.28 [-1.26-0.71]	-0.57
**Freestyle**					
100 m	10.66 ± 0.29	10.62 ± 0.15	0.70	0.19 [-0.79-1.17]	0.38
200 m	11.44 ± 0.14	11.51 ± 0.16	0.43	-0.40 [-1.39-0.60]	-0.61
400 m	11.95 ± 0.12	12.11 ± 0.14	0.03	-1.21 [-2.26- -0.11]	-1.34
800 m	12.20 ± 0.13	12.21 ± 0.16	0.82	-0.12 [-1.10-0.87]	-0.08
**Individual medley**					
200 m	13.17 ± 0.29	13.17 ± 0.23	0.95	0.03 [-0.95-1.01]	0.00
400 m	13.55 ± 0.25	13.76 ± 0.26	0.13	-0.47 [-0.80-0.07]	-1.55

Supplementary Table 5. Comparing breakout distance after turn [m] between the 2016 and 2021 European championships using independent t-test, effect size (ES) with 95% confidence interval (CI), and percent difference (%-diff).

Event	Competition year		*P*	ES [95% CI]	%-diff
	2016	2021			
**Males**					
**Butterfly**					
100 m	10.9 ± 2.3	11.7 ± 1.5	0.46	0.38 [-0.62-1.36]	7.34
200 m	9.0 ± 1.2	9.8 ± 1.3	0.23	0.62 [-0.39-1.62]	8.89
**Backstroke**					
100 m	11.2 ± 1.0	11.5 ± 1.1	0.56	0.30 [-0.70-1.28]	2.68
200 m	9.4 ± 2.2	11.4 ± 2.2	0.09	0.92 [-0.13-1.95]	21.28
**Breaststroke**					
100 m	9.9 ± 0.5	10.5 ± 0.9	0.08	0.97 [-0.11-2.01]	6.06
200 m	10.4 ± 0.7	10.8 ± 1.0	0.42	0.42 [-0.58-1.40]	3.85
**Freestyle**					
100 m	6.9 ± 2.3	8.1 ± 1.7	0.08	0.53 [0.01-0.83]	17.39
200 m	6.0 ± 0.5	7.4 ± 1.3	0.02	1.47 [0.25-2.63]	23.33
400 m	6.0 ± 0.5	6.8 ± 0.5	0.01	1.53 [0.38-2.64]	13.33
1500 m	5.5 ± 1.1	5.1 ± 1.1	0.71	-0.33 [-1.92-1.31]	-7.27
**Individual medley**					
200 m	9.6 ± 0.8	9.6 ± 0.9	0.93	0.05 [-0.94-1.02]	0.00
400 m	8.2 ± 1.2	8.2 ± 0.9	0.96	0.03 [-0.50-0.55]	0.00
**Females**					
**Butterfly**					
100 m	8.2 ± 1.4	9.7 ± 2.2	0.29	0.83 [-0.21-1.84]	18.29
200 m	7.6 ± 1.7	8.0 ± 1.5	0.62	0.25 [-0.74-1.23]	5.26
**Backstroke**					
100 m	10.7 ± 2.2	10.0 ± 2.1	0.50	-0.34 [-1.32-0.65]	-6.54
200 m	7.7 ± 1.5	8.5 ± 1.6	0.32	0.52 [-0.49-1.51]	10.39
**Breaststroke**					
100 m	8.3 ± 0.4	8.7 ± 0.5	0.09	0.90 [-0.15-1.92]	4.82
200 m	8.2 ± 0.3	9.2 ± 0.6	<0.01	2.04 [0.78-3.25]	12.20
**Freestyle**					
100 m	6.3 ± 0.7	7.2 ± 1.3	0.10	0.87 [-0.18-1.88]	14.29
200 m	4.8 ± 0.3	5.4 ± 0.8	0.07	1.03 [-0.07-2.09]	12.50
400 m	4.4 ± 0.8	3.9 ± 0.7	0.23	-0.63 [-1.63-0.38]	-11.36
800 m	4.5 ± 0.6	4.0 ± 0.5	0.15	-0.76 [-1.77-0.27]	-11.11
**Individual medley**					
200 m	6.6 ± 0.8	7.2 ± 1.1	0.19	0.41 [-0.15-0.77]	9.09
400 m	6.4 ± 0.7	6.2 ± 0.4	0.51	-0.33 [-1.32-0.66]	-3.13

Supplementary Table 6. Comparing swimming velocity [m/s] between the 2016 and 2021 European championships using independent t-test, effect size (ES) with 95% confidence interval (CI), and percent difference (%-diff).

Event	Competition year		*P*	ES [95% CI]	%-diff
	2016	2021			
**Males**					
**Butterfly**					
100 m	1.82 ± 0.02	1.84 ± 0.02	0.08	0.95 [-0.10-1.98]	1.10
200 m	1.66 ± 0.02	1.68 ± 0.03	0.08	0.53 [0.01-0.83]	1.20
**Backstroke**					
100 m	1.73 ± 0.01	1.77 ± 0.02	<0.01	1.92 [0.69-3.11]	2.31
200 m	1.60 ± 0.02	1.62 ± 0.03	0.06	1.00 [-0.06-2.04]	1.25
**Breaststroke**					
100 m	1.56 ± 0.03	1.60 ± 0.02	0.02	1.38 [0.26-2.46]	2.56
200 m	1.45 ± 0.02	1.48 ± 0.02	0.01	1.55 [0.39-2.66]	2.07
**Freestyle**					
50 m	2.12 ± 0.02	2.12 ± 0.02	0.45	-0.39 [-1.37-0.61]	0.00
100 m	1.95 ± 0.02	1.98 ± 0.02	0.01	1.55 [0.39-2.66]	1.54
200 m	1.79 ± 0.02	1.81 ± 0.02	0.03	0.66 [0.20-0.88]	1.12
400 m	1.69 ± 0.02	1.70 ± 0.01	0.05	1.05 [-0.02-2.09]	0.59
1500 m	1.65 ± 0.03	1.65 ± 0.01	1.00	0.00 [-1.60-1.60]	0.00
**Individual medley**					
200 m	1.59 ± 0.01	1.63 ± 0.02	<0.01	2.85 [1.39-4.26]	2.52
400 m	1.51 ± 0.02	1.53 ± 0.02	0.02	1.28 [0.17-2.35]	1.32
**Females**					
**Butterfly**					
100 m	1.64 ± 0.03	1.63 ± 0.02	0.35	-0.48 [-1.47-0.52]	-0.61
200 m	1.50 ± 0.02	1.50 ± 0.02	0.89	0.07 [-0.91-1.05]	0.00
**Backstroke**					
100 m	1.57 ± 0.02	1.58 ± 0.04	0.89	0.07 [-0.91-1.05]	0.64
200 m	1.46 ± 0.03	1.48 ± 0.03	0.09	0.90 [-0.14-1.92]	1.37
**Breaststroke**					
100 m	1.40 ± 0.01	1.43 ± 0.01	<0.01	0.88 [0.65-0.96]	2.14
200 m	1.33 ± 0.03	1.34 ± 0.02	0.36	0.28 [-0.29-0.70]	0.75
**Freestyle**					
50 m	1.89 ± 0.03	1.91 ± 0.02	0.21	0.66 [-0.36-1.66]	1.06
100 m	1.75 ± 0.03	1.77 ± 0.02	0.18	0.71 [-0.33-1.73]	1.14
200 m	1.64 ± 0.01	1.64 ± 0.02	0.69	-0.20 [-1.18-0.78]	0.00
400 m	1.56 ± 0.02	1.57 ± 0.02	0.42	0.42 [-0.58-1.40]	0.64
800 m	1.53 ± 0.01	1.53 ± 0.02	0.87	0.08 [-0.90-1.06]	0.00
**Individual medley**					
200 m	1.47 ± 0.02	1.47 ± 0.01	0.46	0.38 [-0.62-1.36]	0.00
400 m	1.38 ± 0.02	1.39 ± 0.02	0.65	0.23 [-0.76-1.21]	0.72

Supplementary Table 7. Comparing stroke rate [bpm] between the 2016 and 2021 European championships using independent t-test, effect size (ES) with 95% confidence interval (CI), and percent difference (%-diff).

Event	Competition year		*P*	ES [95% CI]	%-diff
	2016	2021			
**Males**					
**Butterfly**					
100 m	54.0 ± 2.3	56.1 ± 2.6	0.11	0.86 [-0.19-1.87]	3.89
200 m	49.1 ± 3.2	49.8 ± 1.8	0.61	0.26 [-0.73-1.24]	1.43
**Backstroke**					
100 m	50.4 ± 2.5	49.7 ± 3.4	0.66	-0.22 [-1.20-0.77]	-1.39
200 m	42.5 ± 1.9	41.4 ± 2.5	0.33	-0.50 [-1.49-0.50]	-2.59
**Breaststroke**					
100 m	52.3 ± 4.7	55.2 ± 3.7	0.20	0.68 [-0.34-1.68]	5.54
200 m	36.6 ± 1.6	35.7 ± 3.1	0.45	-0.39 [-1.37-0.61]	-2.46
**Freestyle**					
50 m	64.8 ± 4.6	61.9 ± 1.8	0.14	-0.45 [-0.79-0.09]	-4.48
100 m	52.8 ± 1.3	50.9 ± 2.4	0.07	-0.99 [-2.01-0.07]	-3.60
200 m	44.0 ± 2.2	43.4 ± 2.0	0.54	-0.31 [-1.29-0.68]	-1.36
400 m	42.4 ± 1.7	39.5 ± 2.8	0.02	-1.26 [-2.33- -0.16]	-6.84
1500 m	39.7 ± 5.7	37.8 ± 5.1	0.70	-0.34 [-1.93-1.30]	-4.79
**Individual medley**					
200 m	43.9 ± 1.7	44.3 ± 1.7	0.66	0.22 [-0.77-1.20]	0.91
400 m	41.0 ± 2.0	40.5 ± 1.5	0.59	-0.28 [-1.26-0.71]	-1.22
**Females**					
**Butterfly**					
100 m	55.7 ± 2.6	56.4 ± 3.0	0.65	0.23 [-0.75-1.21]	1.26
200 m	51.4 ± 2.9	51.6 ± 2.5	0.89	0.07 [-0.91-1.05]	0.39
**Backstroke**					
100 m	50.2 ± 3.0	48.1 ± 3.4	0.21	-0.65 [-1.65-0.37]	-4.18
200 m	43.9 ± 2.2	41.1 ± 4.0	0.11	-0.87 [-1.89-0.19]	-6.38
**Breaststroke**					
100 m	47.0 ± 4.1	48.6 ± 6.1	0.55	0.31 [-0.68-1.29]	3.40
200 m	36.5 ± 3.3	35.8 ± 3.8	0.69	-0.21 [-1.18-0.78]	-1.92
**Freestyle**					
50 m	60.8 ± 3.1	60.2 ± 2.9	0.72	-0.18 [-1.16-0.80]	-0.99
100 m	50.5 ± 3.7	49.1 ± 2.8	0.42	-0.42 [-1.40-0.58]	-2.77
200 m	44.8 ± 2.8	43.7 ± 2.8	0.42	-0.41 [-1.40-0.58]	-2.46
400 m	44.0 ± 2.0	45.2 ± 4.8	0.53	0.32 [-0.67-1.30]	2.73
800 m	43.6 ± 2.7	44.8 ± 3.8	0.48	0.36 [-0.63-1.34]	2.75
**Individual medley**					
200 m	45.3 ± 2.7	45.2 ± 2.4	0.96	-0.02 [-1.00-0.96]	-0.22
400 m	42.3 ± 2.7	42.0 ± 2.3	0.84	-0.10 [-1.08-0.88]	-0.71

Supplementary Table 8. Comparing distance per stroke [m] between the 2016 and 2021 European championships using independent t-test, effect size (ES) with 95% confidence interval (CI), and percent difference (%-diff).

Event	Competition year		*P*	ES [95% CI]	%-diff
	2016	2021			
**Males**					
**Butterfly**					
100 m	2.03 ± 0.09	1.98 ± 0.11	0.23	-0.38 [-0.75-0.19]	-2.46
200 m	2.04 ± 0.15	2.03 ± 0.10	0.92	-0.05 [-1.03-0.93]	-0.49
**Backstroke**					
100 m	2.07 ± 0.11	2.15 ± 0.14	0.25	0.60 [-0.41-1.60]	3.86
200 m	2.26 ± 0.11	2.35 ± 0.12	0.12	0.82 [-0.22-1.83]	3.98
**Breaststroke**					
100 m	1.80 ± 0.13	1.75 ± 0.11	0.39	-0.45 [-1.43-0.55]	-2.78
200 m	2.38 ± 0.10	2.51 ± 0.21	0.15	0.76 [-0.27-1.77]	5.46
**Freestyle**					
50 m	1.97 ± 0.12	2.05 ± 0.06	0.19	0.41 [-0.15-0.77]	4.06
100 m	2.22 ± 0.06	2.34 ± 0.10	0.01	1.43 [0.30-2.52]	5.41
200 m	2.44 ± 0.10	2.51 ± 0.12	0.22	0.64 [-0.37-1.64]	2.87
400 m	2.39 ± 0.09	2.60 ± 0.17	0.01	1.47 [0.33-2.57]	8.79
1500 m	2.52 ± 0.33	2.64 ± 0.36	0.67	0.37 [-1.27-1.97]	4.76
**Individual medley**					
200 m	2.17 ± 0.09	2.22 ± 0.10	0.36	0.48 [-0.53-1.46]	2.30
400 m	2.21 ± 0.11	2.27 ± 0.09	0.25	0.60 [-0.41-1.60]	2.71
**Females**					
**Butterfly**					
100 m	1.77 ± 0.10	1.74 ± 0.10	0.47	-0.37 [-1.35-0.63]	-1.69
200 m	1.76 ± 0.11	1.75 ± 0.09	0.89	-0.07 [-1.05-0.91]	-0.57
**Backstroke**					
100 m	1.89 ± 0.11	1.97 ± 0.15	0.21	0.65 [-0.37-1.65]	4.23
200 m	2.00 ± 0.10	2.18 ± 0.20	0.04	1.18 [0.05-2.26]	9.00
**Breaststroke**					
100 m	1.81 ± 0.15	1.79 ± 0.22	0.90	-0.07 [-1.05-0.92]	-1.10
200 m	2.20 ± 0.21	2.27 ± 0.24	0.54	0.32 [-0.67-1.30]	3.18
**Freestyle**					
50 m	1.87 ± 0.09	1.91 ± 0.08	0.44	0.40 [-0.60-1.38]	2.14
100 m	2.09 ± 0.15	2.17 ± 0.13	0.27	0.57 [-0.44-1.56]	3.83
200 m	2.20 ± 0.14	2.25 ± 0.14	0.48	0.36 [-0.64-1.34]	2.27
400 m	2.13 ± 0.10	2.10 ± 0.22	0.71	-0.19 [-1.17-0.80]	-1.41
800 m	2.10 ± 0.13	2.06 ± 0.16	0.52	-0.33 [-1.31-0.66]	-1.90
**Individual medley**					
200 m	1.95 ± 0.11	1.96 ± 0.11	0.85	0.10 [-0.88-1.08]	0.51
400 m	1.97 ± 0.12	1.99 ± 0.09	0.76	0.15 [-0.83-1.13]	1.02
